# IRF2BP2 3′UTR Polymorphism Increases Coronary Artery Calcification in Men

**DOI:** 10.3389/fcvm.2021.687645

**Published:** 2021-10-25

**Authors:** Ragnar O. Vilmundarson, An Duong, Fariborz Soheili, Hsiao-Huei Chen, Alexandre F. R. Stewart

**Affiliations:** ^1^Department of Biochemistry, Microbiology and Immunology, University of Ottawa, Ottawa, ON, Canada; ^2^Laboratory of Translational Genomics, John and Jennifer Ruddy Canadian Cardiovascular Genetics Centre, University of Ottawa Heart Institute, Ottawa, ON, Canada; ^3^Department of Cellular and Molecular Medicine, University of Ottawa, Ottawa, ON, Canada; ^4^The Ottawa Hospital Research Institute, Ottawa, ON, Canada

**Keywords:** coronary artery calcification, ELAVL1/HuR, IRF2BP2, atherosclerosis, genetic variant

## Abstract

Interferon regulatory factor 2 binding protein 2 (IRF2BP2) suppresses the innate inflammatory response of macrophages. A 9-nucleotide deletion (rs3045215) in the 3′ untranslated region (3′-UTR) of human IRF2BP2 mRNA confers risk of coronary artery disease (CAD) in the Ottawa Heart Genomics Study (OHGS). Here, we sought to identify regulatory mechanisms that may contribute to this risk. We tested how lipopolysaccharides (LPS) affects IRF2BP2 expression in human THP-1 macrophages and primary aortic smooth muscle cells (HAoSMC) genotyped for the deletion allele. Both cell types are implicated in coronary atherosclerosis. We also examined how the deletion affects interaction with RNA binding proteins (RBPs) to regulate IRF2BP2 expression. LPS altered allele-specific binding of RBPs in RNA gel shift assays with the THP-1 macrophage protein extracts. The RBP ELAVL1 suppressed the expression of a luciferase reporter carrying the 3′UTR of IRF2BP2 with the deletion allele. Other RBPs AUF1 or KHSRP did not confer such allele specific regulation. Since it is co-inherited with a risk variant for osteoporosis, a condition tied to arterial calcification, we examined the association of the deletion allele with coronary artery calcification in individuals who had undergone computed tomography angiography in the OHGS. In 323 individuals with a minimal burden of atherosclerosis (<30% coronary stenosis) and 138 CAD cases (>50% stenosis), Mendelian randomization revealed that the rs3045215 deletion allele significantly increased coronary artery calcification in men with minimal coronary stenosis. Thus, not only does the rs3045215 deletion allele predict atherosclerosis, but it also predisposes to early-onset calcification in men.

## Introduction

Inflammatory macrophages play a central role in atherosclerosis (1) and are also emerging as an important component of osteoporosis (2). Atherosclerosis and osteoporosis are two conditions that predispose to coronary artery calcification (3, 4). When stimulated with agents such as bacterial lipopolysaccharides (LPS) or viral infection, macrophages acquire an inflammatory M1 phenotype that is thought to hasten the progression of these aging-related diseases. Macrophages respond to LPS by activating the production of type I (5) and type II interferons (6). Induction of interferon regulatory factor 1 (IRF1) plays a key role in the activation of the inflammatory response to LPS (7) by binding to *cis*-regulatory DNA sequences of interferon-responsive genes. Under basal conditions, these interferon-responsive genes are suppressed by competitive binding of the related factor IRF2 that is constitutively expressed (8). IRF2 owes its repressor function to its interaction with IRF2 binding protein 2 (IRF2BP2) (9) that recruits the corepressors NCOR1 (10) and VGLL4 (11).

Ablation of Irf2bp2 in the myeloid lineage by mating mice with a floxed Irf2bp2 allele to LysMCre mice causes macrophages to acquire an inflammatory phenotype (12). Macrophages lacking Irf2bp2 worsened atherosclerosis in murine models due in part to impaired macrophage cholesterol export. Irf2bp2-deficient macrophages have a greater propensity to become laden with triglycerides and cholesterol droplets and to form inflammatory foam cells that are characteristic of atherosclerotic lesions. Irf2bp2 is required for macrophages to achieve the M2 anti-inflammatory phenotype, because Irf2bp2-deficient macrophages fail to activate the M2 program and hyper-activate inflammatory genes when exposed to LPS (12). In the brain of LysMCre/Irfb2bp2^flox^ mice, microglia also display an inflammatory phenotype that delays functional recovery from ischemic brain injury (13, 14) and leads to anxiety-like behaviors in newborn mice (15).

In human macrophages derived from circulating monocytes, IRF2BP2 mRNA levels are reduced in pro-inflammatory M1 macrophages stimulated with LPS compared to anti-inflammatory M2 macrophages (16). Similarly, mouse bone marrow-derived macrophages stimulated with LPS downregulate the Irf2bp2 protein within 8 h (12). We identified a 9-nucleotide deletion variant (rs3045215) in the 3′untranslated region (3′UTR) of human IRF2BP2 mRNA that associates with reduced protein levels of IRF2BP2 in peripheral blood mononuclear cells (12). Since loss of Irf2bp2 in mouse macrophages worsened atherosclerosis, we asked whether this deletion variant associated with coronary artery disease (CAD) in a subset of the OHGS consisting of 1,066 cases of CAD and 1,011 controls. In the OHGS, under a recessive model, having two copies of the deletion allele increased the odds of CAD compared to no or one copy of the deletion allele when adjusting for known risk factors (12).

To determine whether the deletion was sufficient to confer differential expression to the IRF2BP2 mRNA, we constructed luciferase reporters with the entire 3′UTR of IRF2BP2 differing only in the presence or absence of the risk (deletion) allele. The luciferase reporter bearing the deletion risk allele was significantly less active than the reporter without the deletion (12). This difference was not accompanied by a difference in mRNA stability, suggesting the deletion affects the translation efficiency of IRF2BP2, although the exact mechanism underlying this effect was not known (12).

The deletion variant is in a region of the 3′UTR of IRF2BP2 that is highly conserved between species and occurs only in humans among primates. We asked whether the deletion risk allele might disrupt binding or regulation by RNA-binding proteins (RBPs). RBPs are essential for post-transcriptional regulation of mRNAs. RBPs bind to RNA through specific domains and many such domains are well-characterized, such as K homology domains and zinc finger domains, but many previously uncharacterized RBPs with no obvious RNA-binding domains have been uncovered through methods such as interactome capture (17, 18). Since the sequence deleted by the rs3045215 variant is AU rich (AUUAUAACU), RBPs that bind preferentially to AU-rich elements (AREs) were considered, including KHSRP (K homology type splicing regulatory protein), AUF1 (AU-Rich Element RNA Binding Protein 1) and ELAVL1 (Embryonic lethal abnormal visual protein-like 1). KHSRP binds preferentially to AREs in the 3′UTR of Interleukin-8 mRNA leading to mRNA degradation (19, 20). AUF1 regulates IL10 expression as part of the NF-κB pathway (21). When knocked out in mice, absence of AUF1 leads to large-scale induction of pro-inflammatory pathways within macrophages, suggesting an important inhibitory function for this RBP (22). ELAVL1, also known as HuR, binds to mRNAs in response to innate immune activation with LPS and together with a partner RBP called TTP regulates mRNA stability and translation (23). Of interest, ELAVL1 has emerged as an important target of a macrophage-specific long non-coding RNA that contributes to the process of atherosclerosis (24).

More recently, a single nucleotide polymorphism (SNP), rs6672925, that lies just 3′ of the IRF2BP2 gene was found to associate with osteoporosis in two large genome-wide association studies (GWAS) (25, 26). The rs6672925 osteoporosis risk variant is in perfect linkage disequilibrium (LD) with the rs3045215 CAD risk deletion variant (27), meaning the 2 alleles are co-inherited. Osteoporosis is associated with elevated risk of coronary artery disease (3) and predicts the occurrence of advanced coronary artery calcification (4). Since osteoporosis and atherosclerosis share similar disease mechanisms, we asked whether the rs3045215 deletion may be the functional allele accounting for these macrophage-mediated diseases (2, 28). In addition to macrophages, smooth muscle cells also participate in the process of atherosclerosis (29) and coronary calcification (30). Intriguingly, the rs6672925 variant was also found to affect IRF2BP2 mRNA levels in the aortic root, suggesting expression in aortic smooth muscle cells may be affected by the linked rs3045215 deletion allele (31).

Here, we show that the IRF2BP2 3′UTR rs3045215 deletion variant affects IRF2BP2 protein levels in human aortic smooth muscle cells (HAoSMC) and mRNA translation modulated by the RBP ELAVL1. Remarkably, rs3045215 increases coronary artery calcification in men with a minimal burden of atherosclerosis, suggesting that the variant not only contributes to osteoporosis (through its linkage to rs6672925), but also contributes to arterial calcification independent of atherosclerosis.

## Materials and Methods

### Cell Culture—THP-1 and HAoSMC

THP-1 cells, an immortalized human monocyte cell line, were grown in suspension in RPMI 1640 medium (Gibco) supplemented with 10% fetal bovine serum (FBS, Gibco) and penicillin-streptomycin (Pen-Strep, Gibco) at a density of 1 × 10^6^/ml. For macrophage differentiation, THP-1 cells were plated at 2 × 10^5^ cells/ml in PMA-containing medium (phorbol 12-myristate 13-acetate at 1 × 10^−7^ M) for 72 h and then switched to PMA-free medium, rested for 24–48 h prior to use. THP-1 macrophages were found to be HNR for the rs3045215 variant after genotyping with the BsrI/PCR method (12) with DNA isolated by the HotSHOT method (32).

Adult primary HAoSMC (Cell Applications) from healthy human donors were grown in SmGm-2 medium (Lonza, Smooth Muscle Growth Medium-2) with 5% FBS and SingleQuots supplements (Lonza, human epidermal growth factor, human fibroblastic growth factor, insulin, and gentamicin/amphotericin-B). Cells were split at 80% confluence using Trypsin-EDTA (Gibco, 0.05%, containing phenol red) and medium was renewed every 2 days. DNA was extracted using the HotSHOT method (32). HAoSMC rs3045215 genotypes were as follows: homozygous non-risk (non-deletion), HNR = 1,441, 1,473 and 3,003; heterozygous, HET = 1,596 and 2,228. No homozygous risk (deleted) HR HAoSMC was identified by genotyping.

### Luciferase Reporter Assays With RBP Co-transfection

The empty control mammalian expression plasmid pReceiver-M02 (#EX-NEG-M02-B), and plasmid vectors for AUF1 (#EX-Z0257-M02-B), ELAVL1 (#EX-Q0365-M02-B), and KHSRP (#EX-Z9878-M02-B) were purchased from Genecopoeia. These vectors were co-transfected with non-risk (rs3045215 non-deletion allele) and risk (rs3045215 deletion allele) luciferase reporters in HEK293 cells using the Lipofectamine 3000 kit (Invitrogen). The luciferase reporters were described previously (12). HEK293 cells were plated at 1 × 10^6^/ml, 1 ml per well, in 12-well-plates and allowed to adhere overnight. The next day, cells were transfected with 1 μg of luciferase reporter (either non-deletion or deleted) in triplicate together with the empty pEZ vector control or with 10, 50, or 100 ng of each RBP vector (either AUF1, KHSRP, or ELAVL1). Each experiment was repeated 5–7 times. Overexpression of each vector in HEK293 cells was confirmed by immunoblot. HEK293 cells were grown in 6-well-plates and maintained in DMEM (low glucose, Gibco) medium with 10% FBS and Pen-Strep.

### LPS Treatment of THP-1 Macrophages and HAoSMC

THP-1 macrophages were treated with LPS (either 10 or 100 ng/ml) to activate the M1 pro-inflammatory state. For immunoblots, protein was extracted at baseline and 4, 8, 24, 48, and 72 h after LPS treatment. The cells were in LPS-containing medium for the entire time until they were harvested (except for baseline cells which were not treated). Baseline controls were harvested at the earliest LPS treatment time point. For cytoplasmic lysis buffer (1% Triton, 25 mM Tris-HCL pH 7.4, 40 mM KCL) and M-PER (Mammalian Protein Extraction Reagent, Thermo Fisher Scientific) whole-cell extracts were acquired at baseline, 4, 8, and 24 h and used in RNA-gel shift experiments.

HAoSMC were treated with LPS (10 ng/ml) for 8, 24, or 48 h prior to RIPA buffer extraction and lysates were used for immunoblots. Cells were seeded to be 50% confluent at the time of LPS treatment. As with THP-1 macrophages, except for baseline controls, HAoSMCS were in LPS-medium for the entire duration. Medium was not changed after start of LPS treatment. For RNA gel shifts, cytoplasmic and M-PER whole-cell extracts were obtained at baseline and 24 h after LPS, based on the THP-1 and HAoSMC immunoblot results.

### Immunoblot Analysis

Proteins were size fractionated by SDS polyacrylamide gel electrophoresis followed by electroblotting to PVDF membranes. Proteins were visualized with the following antibodies: a rabbit peptide-specific antibody to IRF2BP2, described previously (11), anti-ELAVL1 mouse monoclonal (Abcam, #ab136542), anti-KHSRP mouse polyclonal (Abnova Corporation, #H00008570-A01), anti-AUF1 rabbit polyclonal (Abcam, #ab50692), anti-alpha-smooth muscle actin mouse monoclonal (Sigma, #A2547), anti-GAPDH mouse monoclonal (Santa Cruz Biotechnology, #sc-59540), and anti-β-actin mouse monoclonal (Sigma, #A2228). Primary antibodies were used at 1/5,000 dilution. The secondary horseradish peroxidase-conjugated antibodies used at a 1/10,000 dilution, goat anti-mouse IgG antibody (R&D Systems, #HAF007) and goat anti-rabbit IgG (H+L) antibody (Life Technologies, #31460), were revealed by chemiluminescence using the SuperSignal West Dura substrate (Thermo Fisher Scientific). Bands were quantified using the ImageJ software (33) with each lane signal normalized to loading control prior to fold conversion. Fold change compared to average of baseline values.

### Electrophoresis Mobility Shift Assay

An RNA EMSA method was used to determine binding differences between risk and non-risk RNA probes. RNA probes (Non-deletion probe, 5′-UAGGCACUUUAUUAUAACUGGAAUUUGAC-3′; Deleted probe, 5′-UAGGCACUUUGGAAUUUGAC-3′) were synthesized by Integrated DNA Technologies. The non-deletion (non-risk) probe included the 9-nucleotide deletion sequence plus 10 bases of flanking sequence on either side. The deleted probe contained only the flanking sequence (20 nucleotides total). Probes containing either the non-deletion or deletion allele of rs3045215 were 5′-end labeled using [^32^P]γ-ATP and T4 polynucleotide kinase at 37°C for 1 h, as described previously (34). The reaction was stopped using 0.5 M EDTA (pH 8.0) and then the samples were purified using RNase-free G-25 Sephadex spin columns (Roche). The following cocktails were prepared for non-deletion and deleted probes separately: 10X binding buffer, 1 μL; Radiolabeled probe (50,000 CPM dilution), 1 μL; 50% glycerol, 1.5 μL; 1 M DTT, 0.2 μL; RNasin (Promega), 0.2 μL; Yeast Total RNA (10 mg/ml), 0.2 μL; 5% xylene cyanol, 1 μL; DEPC-treated ddH20, 2.9 μL. All components were prepared or purchased RNase-free. Binding buffer composition can be found in the Yakhnin et al. (35) reference under Csra binding buffer 10X. Cocktails were added last to tubes containing protein extracts, gently vortexed, spun down and incubated at 37°C for 30 min. For DNA gel shifts, a double-stranded NF-κB-specific oligonucleotide probe (sense 5′-AGA*GGGGACTTTCC*GAGG-3′) was synthesized by IDT Technologies. DNA EMSA were carried out as described previously (36). Samples were run on a 7% poly-acrylamide gel for 3 h at 200 volts in a cold room. Gels were dried before being placed in a cassette with a phosphor screen overnight at room temperature and the image was captured the next day using a Storm phosphor imaging system (GE Healthcare Lifesciences). Quantification for EMSA experiments was performed using ImageQuant software (V2.0, Cytiva Lifesciences, formerly GE Healthcare Lifesciences). The probe alone lane, where no shifted complex was present, was used to subtract background values from all lanes.

### Genotyping Rs3045215 in OHGS CT Angiography Samples

The OHGS cohort consists of 3,273 individuals of mixed European ancestry who have undergone coronary angiography and are defined as early onset cases of coronary artery disease (CAD, with >50% stenosis in a major coronary artery, before 55 years in males and 65 years in females) and 1,066 asymptomatic elderly controls (>65 years of age for males, >70 years of age for females) recruited from the Ottawa region who have undergone genome-wide genotyping of SNPs (37, 38). The OHGS excluded individuals with diabetes. For the subset of individuals who were recruited from CT angiography for which coronary calcium Agatston scores are available, the age restrictions were relaxed by 10 years to include 323 controls who displayed minimal coronary stenosis (<30% stenosis in a major coronary artery) and 138 CAD cases (>50% stenosis in a major coronary). The study (protocol number 2009857) was approved by the Ottawa Health Science Network Research Ethics Board (OHSN-REB), conformed to the principles outlined in the Declaration of Helsinki and all participants gave written informed consent to participate in the study. Because the rs3045215 variant is not detected on Affymetrix arrays used to genotype the OHGS cohort, DNA samples were genotyped using a BsrI restriction enzyme digest of a PCR amplicon of the rs304515 sequence, as described previously (12). Genotypes did not deviate from Hardy-Weinberg equilibrium.

### Statistical Analysis

All statistical analyses were carried out using the R statistical computing software (https://www.R-project.org). For all transfection experiments, luciferase reporter activity levels were normalized as log_2_ fold change relative to the non-deletion construct baseline average. To determine if co-transfection produced a dose-dependent effect and whether there was an interaction between dose and vector type (non-deletion/deletion luciferase reporters) a two-way analysis of variance (ANOVA) was performed for each co-transfection experiment. Dose, luciferase reporter type plus a dose:vector interaction variable were included in each ANOVA. *P* ≤ 0.05 were considered significant. Tukey's *post-hoc* analysis was performed when there was significant interaction between vector type and dose.

To determine the association of the rs3045215 deletion with coronary artery calcification a linear regression model was used. The rs3045215 variant was coded as counts of the minor (deletion) allele, i.e., HNR = 0, HET = 1, and HR = 2. The analysis was split by sex and CAD case status with smoking status and age included in the linear model. Agatston scores were normalized using log_2_ after adding 1 to address zero values in the data. Linear regression was performed using the lm function in R and data was plotted using the data visualization package ggplot2 (39).

## Results

### LPS Modulation of IRF2BP2 Expression Is Cell and Allele-Specific

**Figure 1:**
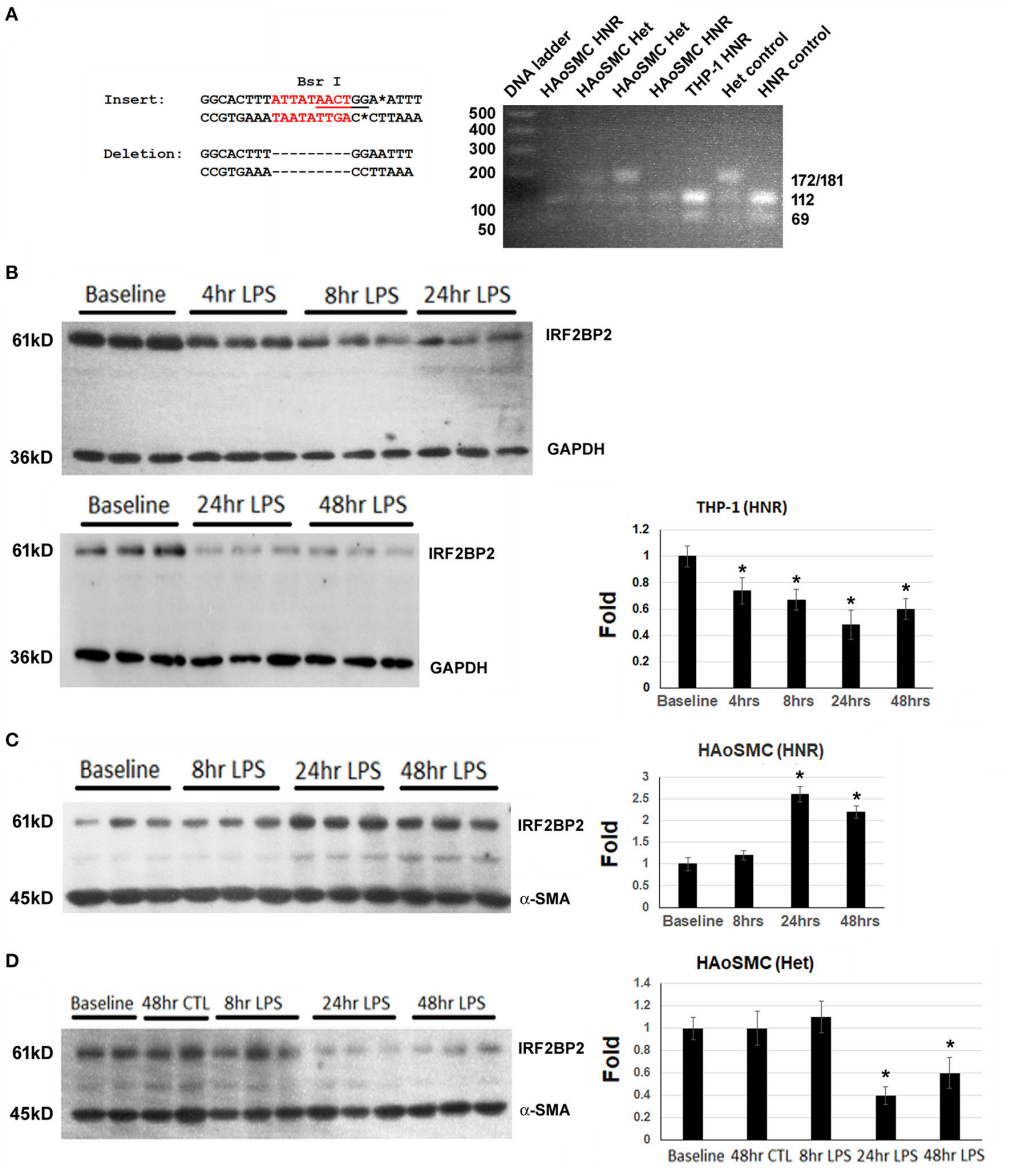
Cell type-specific and allele-specific regulation of IRF2BP2 in response to innate immune activation. **(A)** BsrI restriction fragment length polymorphism (RFLP) genotyping of genomic DNA from THP-1 cells and Human aortic smooth muscle cells (HAoSMC). The insertion sequence contains the BsrI site, while the deletion allele does not. HNR, homozygous non-risk; Het, heterozygous. **(B)** Human THP-1 macrophage cells homozygous for the non-risk allele (HNR, non-deletion allele of rs3045215) down-regulate IRF2BP2 protein in response to LPS. **(C)** Human aortic smooth muscle cells (HAoSMC) from HNR donors upregulate IRF2BP2 in response to LPS (*n* = 3 donors). **(D)** HAoSMC from heterozygote donors down-regulate IRF2BP2 protein levels in response to LPS (*n* = 2 donors). Representative blots are shown. Bands were quantified by densitometry and normalized to GAPDH for THP-1 blots and alpha smooth muscle actin for HAoSMC blots. Values are fold change relative to average of baseline values. Cells were treated with 10ng/ml LPS. *p < 0.05 from baseline by Student's *t*-test.

THP-1 macrophages were genotyped and found to be homozygous for the non-risk (HNR) (i.e., 2 copies of the non-deletion) allele of rs3045215 (Figure 1A). THP-1 macrophages showed a rapid time-dependent reduction in IRF2BP2 protein levels in response to LPS (Figure 1B). Primary human aortic smooth muscle cells (HAoSMC) from 5 donors were also genotyped and 3 were found to be HNR and 2 were heterozygotes (HET, carrying 1 copy of the non-deletion allele and 1 copy of the 9-nucleotide deletion allele). We were unable to detect HAoSMC from any donor homozygous for the risk allele, due to the low minor allele frequency. In contrast to THP-1 cells, HAoSMC from HNR donors showed a delayed increase in IRF2BP2 levels in response to LPS challenge (Figure 1C). Surprisingly, HAoSMC from HET donors showed a delayed reduction in IRF2BP2 expression with LPS treatment (Figure 1D).

### LPS Alters Allele-Specific and Cell-Specific Binding of RBPs

**Figure 2:**
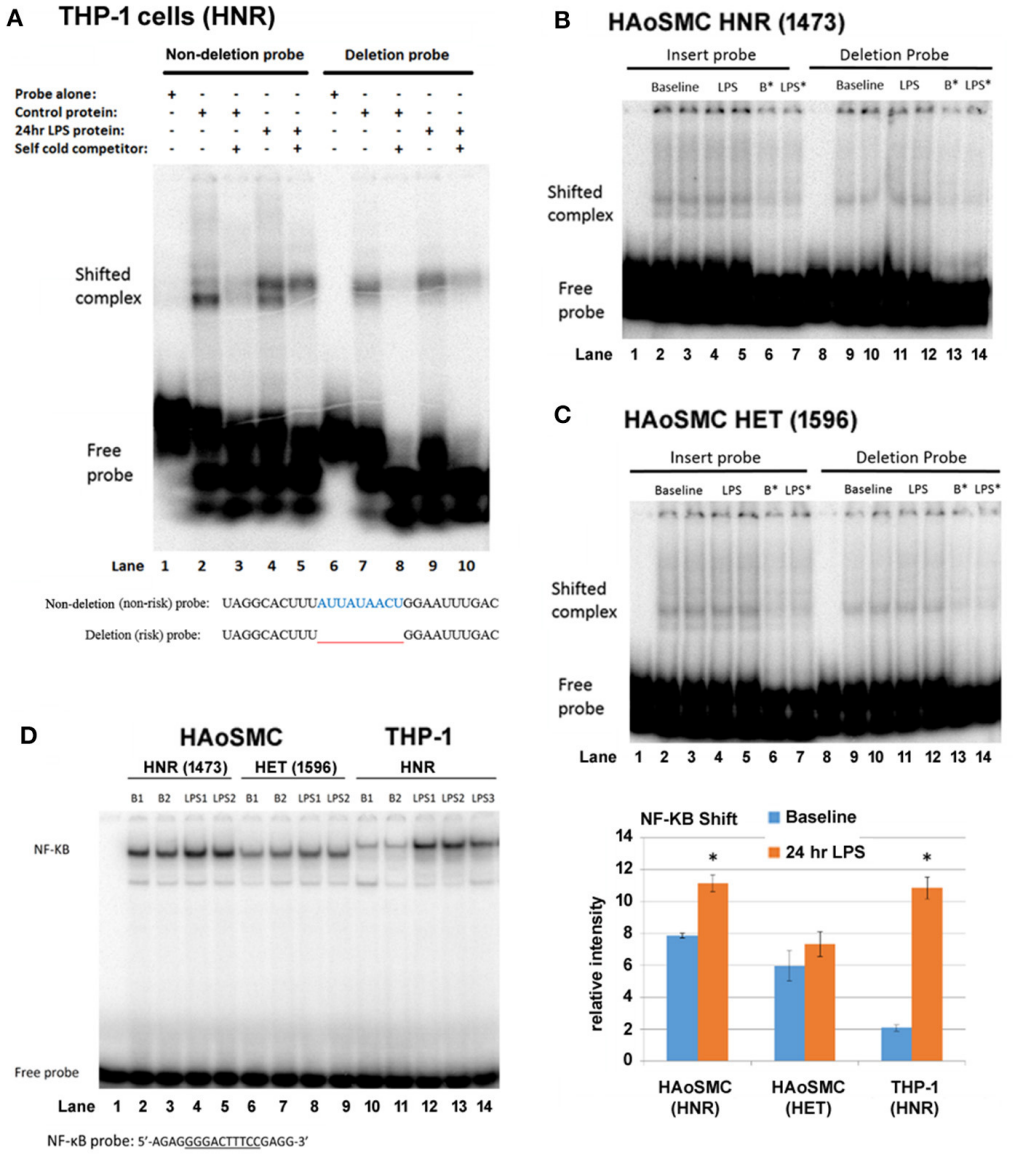
THP-1 macrophages, but not HAoSMC, show differential complex formation between non-deletion (non-risk) and deleted (risk) probes after LPS treatment. **(A)** Representative RNA gel shifts of cytoplasmic RNA binding proteins from control or LPS-stimulated THP-1 macrophages. The sequence of the radiolabeled non-deletion (non-risk) and deleted (risk) single-stranded RNA probes is indicated below the autoradiograph. Lanes 1/6 are probes alone, lanes 2/7 are with control THP-1 extract, lanes 3 and 8 are with 1,000-fold cold probe competitor, lanes 4/9 are with LPS-treated THP-1 extract and lanes 5/10 are with the addition of cold competitor. LPS (100 ng/ml). Note the appearance of a lower mobility (upper) shifted complex with LPS treatment only for the non-deletion (non-risk) probe. **(B)** Representative RNA gel shifts of cytoplasmic RNA binding proteins from control and LPS-treated HAoSMC from an HNR donor (1,473, top panel) and **(C)** a HET donor (1596, bottom panel). Non-deletion probe shows a doublet while the deleted probe shows a single shifted complex. LPS treatment has no visible effect on the intensity of shifted complexes. B* and LPS*, baseline or LPS + 1,000-fold cold competitor. **(D)** NF-κB DNA gel shifts were used as a positive control for the LPS response. Nuclear proteins from HAoSMC from an HNR donor (1,473), from HAoSMC of a HET donor (1,596) and from THP-1 human macrophage cells were tested for binding to an NF-κB specific double-stranded DNA probe (only the upper strand is shown). Phosphorimager quantitation of shifted complexes reveals a weak LPS response in HAoSMC compared to a robust LPS response in THP-1 macrophages. *p < 0.05 compared to own baseline by Student's *t*-test.

We asked whether the region of the 3′UTR of IRF2BP2 containing the deletion showed any allele-specific binding of RBPs that could account for allele-specific regulation of IRF2P2 protein levels. RNA gel shifts were carried out using protein extracts from THP-1 macrophages tested on radiolabeled RNA probes carrying either the non-deletion or deletion alleles. The effect of LPS on RBP binding to these two alleles was also compared. The radiolabeled RNA probe of the non-deletion allele revealed 2 shifted complexes, a robust lower fast mobility complex and a weaker higher slower mobility complex, using cytosolic proteins from untreated THP-1 macrophages (Figure 2A, lane 2). In contrast, the RNA probe carrying the deletion allele revealed a single shifted complex (Figure 2A, lane 7). Competition with 1000-fold excess of non-radiolabeled cold RNA probes showed all these complexes were specific (Figure 2A, lanes 3 and 8). LPS-treatment changed the distribution of the shifted complexes with the non-deletion probe: the slower mobility upper complex became predominant (Figure 2A, lane 4). On the other hand, LPS treatment increased binding to the single shifted complex formed with the deletion probe (Figure 2A, lane 9). These RNA gel shift results reveal an allele-specific effect on RBP interaction with the IRF2BP2 mRNA. Moreover, the effect of LPS on RBP binding to IRF2BP2 mRNA is lost with the deletion (risk) allele.

Next, we tested whether an allele-specific effect on RNA binding in response to LPS treatment could be observed in cytosolic extracts of HAoSMC from donors HNR and HET for rs3045215 (Figures 2B,C). As in THP-1 macrophages, HAoSMC of either genotype showed allele-specific differences in RNA binding complexes, with 2 complexes formed with the non-deletion probe and a single complex formed with the deletion probe. The lower mobility complex (whose binding increases in THP-1 cells with LPS treatment) was more abundant relative to the higher mobility complex in HAoSMC at baseline. However, HAoSMC did not show increased binding of this complex in response to LPS treatment.

Concerned that HAoSMC might not respond to LPS treatment to the same extent as THP-1 macrophages, nuclear protein extracts were tested for changes in NF-κB binding in DNA gel shift assays. Indeed, in contrast to THP-1 macrophages that showed a robust increase in NF-κB binding in response to LPS treatment (Figure 2D, compare lanes 10/11 to 12–14), HAoSMC for a HNR or HET donors showed a much smaller increase in binding to the NF-κB probe (Figure 2D, compare lanes 2/3 to 4/5, and lanes 6/7 to 8/9). Importantly, while these cells are of human origin, the size of the shifted complexes is clearly different between HAoSMC and THP-1 macrophages, suggesting different isoforms of NF-κB are expressed in these 2 cell types. Also, baseline levels of the NF-κB complex were much more abundant in untreated HAoSMC than in THP-1 macrophages. Thus, primary HAoSMC may already be LPS “primed” compared to THP-1 macrophages.

### ELAVL1 Elicits Allele-Specific Suppression of Protein Expression

Given that the deletion allele affects RBP interaction with the 3'UTR of IRF2BP2 mRNA, and that luciferase reporters carrying the entire 3'UTR of human IRF2BP2 but differing only in the presence or absence of the rs3045215 deletion showed different expression, we sought to identify RBPs involved in suppressing the expression of IRFPBP2 deletion variant.

**Figure 3:**
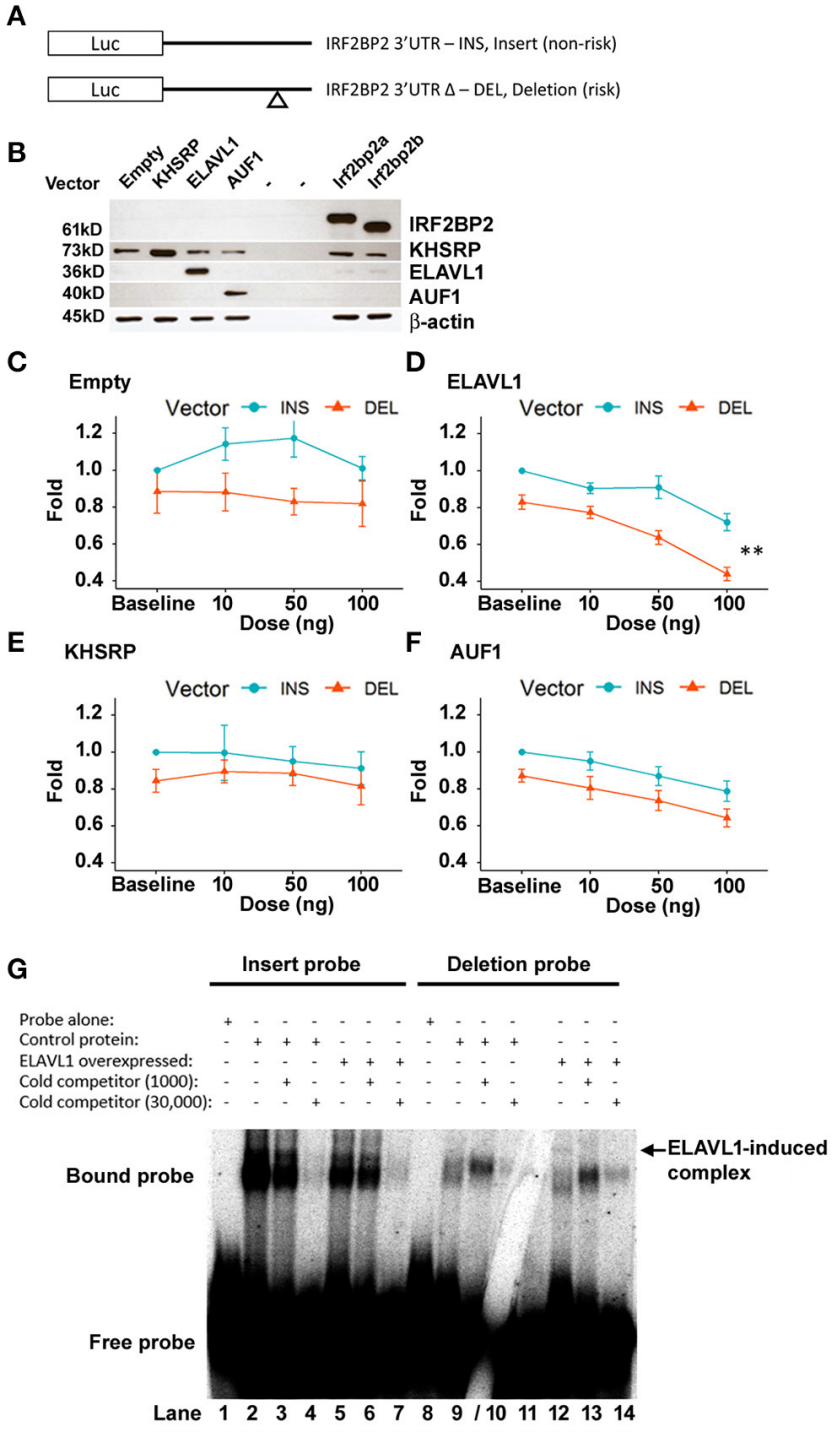
The deletion variant confers ELAVL1-dependent suppression of luciferase reporter activity. **(A)** CMV-driven luciferase reporter plasmids bearing the 3′UTR sequence of IRF2BP2 and differing only in the presence of the non-risk allele (pCMV-i-3′UTR-IRF2BP2, INS, non-risk) or the risk allele (pCMV-Δ-3′UTR-IRF2BP2, DEL, risk) of rs3045215 were transfected into human HEK293 cells with either empty expression vector (*N* = 5), KHSRP (*N* = 5), AUF1 (*N* = 7), or ELAVL1 (HuR) (*N* = 7). **(B)** HEK293 whole cell lysates show similar levels of over-expressed RBP after transfection with each expression plasmid. Plasmids expressing the IRF2BP2a and IRF2BP2b splicing isoforms were used as transfection controls. β-actin served as a loading control. Activity of luciferase reporters was compared when co-transfected with various doses of expression plasmids: **(C)** empty expression plasmid, **(D)** ELAVL1, **(E)** KHSRP, and **(F)** AUF1. The luciferase reporter activity was expressed as a normalized fold relative to non-deletion luciferase reporter activity at baseline. **Interaction between ELAVL1 and luciferase reporters [two-way ANOVA, *F*_(3, 48)_ = 4.322, *p* < 0.0089]. **(G)** ELAVL1 protein forms a new specific complex with the deletion probe (lane 12, arrow). Cytoplasmic protein extracts from HEK293 cells without and with ELAVL1 overexpression by transient transfection. Cold competitor used at 1,000- and 30,000-fold of non-deletion (lanes 3, 4) and deletion (lanes 9/10) RNA probes, respectively. Gel crack through 30,000-fold cold competitor for deleted probe does not affect overall result.

HEK293 cells were co-transfected with either of these two IRF2BP2-luciferase reporters (diagram, Figure 3A) together with expression plasmids for various RBPs. Since the deleted sequence contains an AU-rich element, we focused on RBPs that target AU-rich sequences, including KHSRP, AUF1, and ELAVL1. Immunoblot showed similar levels of over-expression of each RBP was achieved (Figure 3B). Luciferase activities were reported as fold of baseline for the non-deletion reporter (Figures 3C–F).

As we reported previously (12), the luciferase reporter bearing the 3′UTR deletion allele had an intrinsically lower luciferase reporter activity (Figure 3C). In cells co-transfected with the empty expression plasmid, two-way ANOVA revealed a significant effect of the luciferase reporter vector [*F*_(1, 32)_ = 1.367, *p* = 0.0011], but no dose effect of the empty expression plasmid [*F*_(3, 32)_ = 0.471, *p* = 0.7043] nor an interaction between expression plasmid dose and the reporter plasmid [*F*_(3, 32)_ = 0.276, *p* = 0.8421].

ELAVL1 expression dose-dependently suppressed both luciferase reporters [*F*_(3, 48)_ = 32.8, *p* <1.12e^−11^; Figure 3D). Importantly, the effect was much more prevalent for the reporter carrying the 3′UTR deletion allele; interaction between the luciferase reporter plasmid and the ELAVL1 expression plasmid dose [*F*_(3, 48)_ = 4.322, *p* = 0.0089]. The difference between luciferase reporters remained highly significant [*F*_(1, 48)_ = 64.2, *p* <2.10e^−10^]. Thus, ELAVL1 may contribute to selective suppression of the IRF2BP2 deletion allele.

In contrast, the KHSRP expression plasmid had no suppressive effect on either luciferase reporter (Figure 3E), indicating that KHSRP is unlikely to directly regulate IRF2BP2 expression; no effect of KHSRP dose [*F*_(3, 32)_ = 0.340, *p* = 0.797] or any interaction between KHSRP plasmid dose or the reporter plasmids [*F*_(3, 32)_ = 0.154, *p* = 0.927] was observed. Surprisingly, with the over-expression of KHSRP, the difference between the two luciferase reporters was no longer significant [*F*_(1, 32)_ = 2.379, *p* = 0.133], suggesting that over-expression of KHSRP may overwhelm the endogenous mechanism conferring the allele-specific difference in IRF2BP2 expression.

AUF1 indiscriminately suppressed both luciferase reporters in dose-dependent manner [F_(3, 48)_ = 8.48, *p* = 0.00013; Figure 3F]; no interaction between AUF1 expression plasmid dose and the reporters was observed [*F*_(3, 48)_ = 0.090, *p* = 0.965]. The difference between luciferase reporters remained significant with AUF1 over-expression [*F*_(1, 48)_ = 16.98, *p* = 0.00015]. Thus, AUF1 downregulates IRF2BP2 expression, and this effect is independent of the deletion allele.

### ELAVL1 Produces a New Specific Complex With the Deletion Allele RNA Probe

We next asked whether the differential effect of ELAVL1 would be reflected in an RNA gel shift assay using the non-deletion and deleted RNA probes. Overexpression of ELAVL1 in HEK293 cells did not change the RNA complexes formed with the non-deletion probe compared to non-transfected cells (Figure 3G, lanes 2 and 5), but induced the formation of a new complex with the deletion RNA probe (Figure 3G, compare lane 8–12, arrow). This new complex was completely competed by 1,000-fold excess of cold probe, demonstrating this binding is specific to the deletion allele (Figure 3G, lane 13). Of note, while the 9-nucleotide deletion removes an AU-rich sequence, the remaining flanking sequence of the deletion RNA probe still contains AU-rich sequences that could bind to ELAVL1.

### IRF2BP2 Deletion (Risk) Allele Associates With Coronary Artery Calcification in Men

To determine whether the deletion allele of rs3045215 contributes to coronary artery calcification, we examined the distribution of this variant in individuals of the OHGS (37) who had undergone CT angiography to assess the degree of coronary artery stenosis and coronary artery calcification, quantified by Agatston scores (40). According to the criteria used in the OHGS, cases with coronary artery atherosclerosis were defined as having >50% stenosis in any major coronary artery and controls as having <30% stenosis. As shown in Table 1, we genotyped rs3045215 in 138 CAD cases and 323 controls and found an association of the minor allele with atherosclerosis (*p* = 0.044), consistent with our prior report (12).

**Table 1:** Clinical characteristics of OHGS CT angiography samples with Agatston scores genotyped for rs3045215.

**Variable**	**Controls (*N* = 323)**	**Cases (*N* = 138)**	***p***
Agatston score, HU	0.00 [0.00, 82.50]	344.00 [121.00, 601.75]	<0.001
Age, years	58.63 (11.05)	62.03 (8.26)	0.001
Male sex, *N* (%)	168 (52.0)	89 (64.5)	0.018
BMI, kg/m^2^	28.35 (5.30)	28.53 (4.94)	0.732
Smoking, *N* (%)	177 (54.8)	100 (72.5)	0.001
HTN, *N* (%)	112 (34.7)	76 (55.1)	<0.001
Total-C, mmol/L	5.69 (1.16)	6.15 (1.19)	0.001
LDL-C, mmol/L	3.57 (0.97)	3.96 (0.85)	0.001
HDL-C, mmol/L	1.37 (0.37)	1.28 (0.35)	0.026
TG, mmol/L	1.40 [0.95, 2.03]	1.65 [1.19, 2.34]	0.004
Statins, *N* (%)	121 (37.5)	79 (57.2)	<0.001
**rs3045215 (%)**			0.044
0	164 (50.8)	59 (42.8)	
1	138 (42.7)	61 (44.2)	
2	21 (6.5)	18 (13.0)	

*Agatston score and triglycerides have non-normal distribution and are therefore given as median with interquartile range (IQR). All other continuous variables are given as mean ± SD. Cholesterol and triglyceride measurements are baseline values. Abbreviations: SD, standard deviation; HU, Hounsfield units; HTN, anti-hypertensive medication; Total-C, total-cholesterol; LDL-C, low density lipoprotein-cholesterol; HDL-C, high density lipoprotein-cholesterol; TG, triglycerides. rs3045215 genotypes are counts of the minor (deletion) allele. Categorical variables were tested with the Chi-square test, continuous variables with the Student's t-test when normally distributed and the Wilcoxon test when not normally distributed*.

We next examined the association of the deletion allele with coronary artery calcification. Crude analysis revealed a significant association of the deletion allele with coronary artery calcification, measured as elevated Agatston scores (Table 2, *p* = 2.17e^−04^). When split by sex, the association of the deletion allele remained significant only in males (*p* = 0.00331). We next carried out regression analysis, considering CAD status, age at consent, sex, and smoking history as covariates in all CT samples together. The deletion allele still associated with the Agatston score (*p* = 0.0448), but CAD status (*p* <2e^−16^), sex (*p* <1.06e^−7^), age at consent (*p* = 2e^−16^), and smoking (*p* = 0.016) also contributed significant effects on Agatston scores.

**Table 2:** Regression analysis of rs3045215 minor allele effect on coronary artery calcification.

**Analysis**		**Variable**	**Beta**	**SE**	**p**	**Risk allele *p* ≤ 0.05**
Crude		rs3045215	1.03	0.277	2.17e−04	*
Crude, split by sex	Males	rs3045215	1.06	0.359	0.00331	*
	Females	rs3045215	0.741	0.422	0.0803	
Risk factor adjusted, including CAD in model		rs3045215	0.418	0.208	0.0448	*
		CAD	4.01	0.298	<2e−16	
		sex	1.47	0.271	1.06e−07	
		age	0.129	0.0131	<2e−16	
		smoking	0.659	0.272	0.016	
Risk factor adjusted, split by CAD case/control	Controls	rs3045215	0.704	0.274	0.0106	*
		Sex	1.613	0.339	2.90e−06	
		Age	0.134	0.0153	<2e−16	
		Smoking	0.849	0.335	0.0117	
	Cases	rs3045215	−0.124	0.288	0.666	
		sex	0.915	0.430	0.0354	
		age	0.0961	0.0258	2.95e−04	
		smoking	−0.0725	0.448	0.872	
Controls only, split by sex	Males	rs3045215	0.984	0.374	0.00934	*
		age	0.155	0.0219	3.99e−11	
		smoking	0.805	0.473	0.0911	
	Females	rs3045215	0.382	0.403	0.344	
		age	0.113	0.0222	1.09e−06	
		smoking	0.758	0.493	0.126	

**Figure 4:**
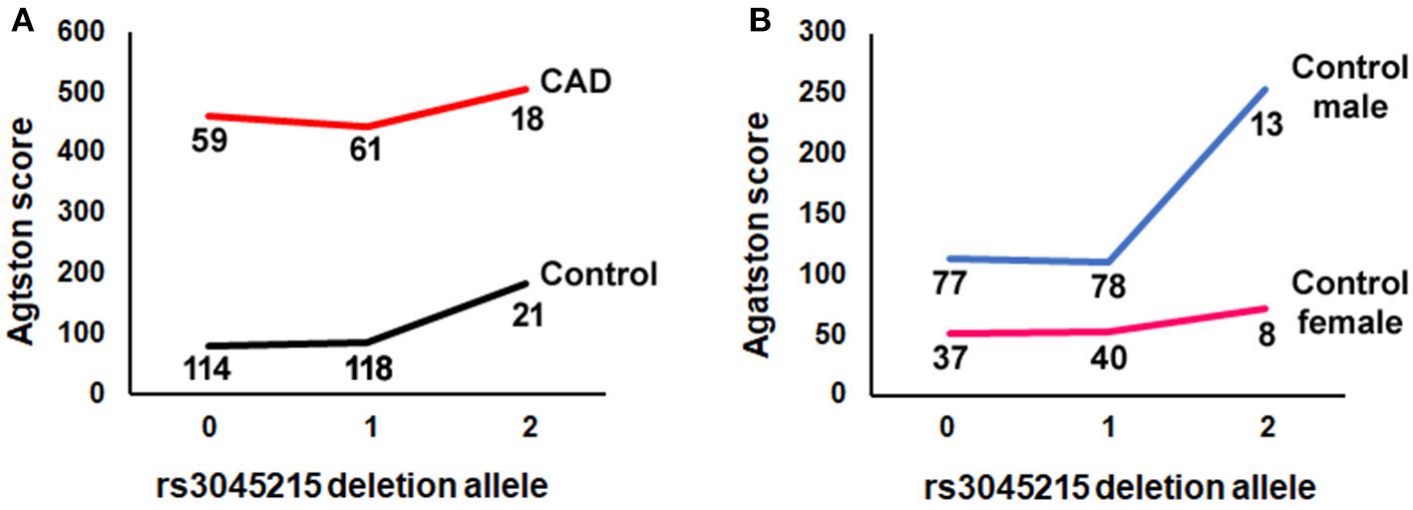
The minor (deletion) allele of rs3045215 associates with coronary artery calcification in men with minimal atherosclerotic burden. **(A)** Agatston score by number of rs3045215 deletion alleles (0, homozygous for the non-deletion allele; 1, heterozygous; 2, homozygous for the deletion allele) in CAD cases and controls. **(B)** Agatston score by number of rs3045215 deletion alleles in male and female controls. Numbers below lines are genotyped individuals.

We also examined whether the association of the deletion allele with calcification was affected by the degree of atherosclerosis, by repeating the regression analysis in CAD cases separately from controls (i.e., Mendelian randomization controlling for the effect of CAD). Unexpectedly, the deletion allele of rs3045215 showed a robust association with Agatston score in controls (*p* = 0.0106), but not in CAD cases (Table 2). Further, we tested whether the effect was sex-dependent by repeating the regression analysis separately in male and female controls. The deletion allele showed a strong association with coronary calcification in males with a minimal burden of atherosclerosis (*p* = 0.00934). The distribution of the rs3045215 genotypes among individuals with minimal coronary disease revealed a strong effect on calcification (with an Agatston score of 254) in men homozygous for the deletion allele, compared to men carrying one or no copy of the deletion (with Agatston scores of 110 and 113) (Figure 4).

## Discussion

Here, we examined the effect of the rs3045215 deletion allele in the 3'UTR of IRF2BP2 on the modulation of IRF2BP2 protein expression by LPS in HAoSMC. We found that HAoSMC that carry 1 copy of the deletion allele down-regulate IRF2BP2 when stimulated with LPS. In contrast, HAoSMC that carry 2 copies of the non-deletion allele upregulate IRF2BP2 with LPS treatment. Thus, the rs3045215 deletion confers a differential response to LPS stimulation in HAoSMCs and may increase their inflammatory response. We also found that human THP-1 macrophages that carry 2 copies of the non-deletion allele downregulate IRF2BP2 with LPS treatment, further revealing a cell type-specific regulation of IRF2BP2 expression by LPS. RNA probes containing the deletion and non-deletion alleles displayed differential binding to RBPs in protein extracts from THP-1 macrophages and HAoSMC. Among candidate RBPs tested, the AU-rich element binding protein ELAVL1 specifically suppressed expression of a luciferase reporter bearing the deletion allele in the 3′UTR of IRF2BP2. RNA gel shift revealed this protein makes a specific complex with the deletion but not the non-deletion RNA probe. Lastly, we found that men homozygous for the rs3045215 deletion allele with a minimal burden of coronary atherosclerosis are susceptible to coronary artery calcification.

IRF2BP2 expression is suppressed in THP-1 macrophages stimulated with LPS, and this augments their innate inflammatory response (12). THP-1 macrophages are homozygous for the non-deletion allele. In contrast, we found HAoSMC homozygous for the non-deletion allele upregulate IRF2BP2 when stimulated with LPS. A similar LPS-induced elevation in IRF2BP2 in the heart of mice has been reported in a study of sepsis-induced cardiomyopathy (41). In this study, IRF2BP2 was found to protect the heart from LPS-induced inflammation and cardiomyopathy. HAoSMC carrying one copy of the rs3045215 deletion allele displayed reduced IRF2BP2 expression in response to LPS. Individuals carrying the deletion allele of rs3045215 may have more inflammatory vascular smooth muscle cells in the presence of atherosclerosis, accounting for the associated increased CAD risk (12). Our observation is consistent with a study of eQTL (expressed quantitative trait loci) SNPs that found lower expression of IRF2BP2 in the human aortic root in individuals who carry the rs6672925 allele (31) that is coinherited with the rs3045215 deletion allele.

Our previous study found an association of the rs3045215 deletion allele with coronary artery disease in individuals who underwent standard coronary angiography for early onset coronary artery disease and asymptomatic population controls (12). Here, using a CT angiography subgroup of the OHGS that we had not genotyped previously, we were able to replicate the association of rs3045215 deletion allele with coronary artery disease. Importantly, this CT angiography cohort provided coronary artery calcification scores that enabled us to determine whether the rs3045215 deletion allele affects calcification. Considering recent reports that a nearby co-inherited SNP was tied to osteoporosis and the link between osteoporosis and vascular calcification, here we found that the minor deletion allele of rs3045215 showed a more robust association with coronary calcification in men with <30% stenosis of any major coronary artery. It is also important to note that a single copy of the deletion allele did not elevate the calcium score above the level in carriers of the non-deletion ancestral allele (see Figure 4). This result is also consistent with our prior report on the association with CAD being more robust in a recessive model than in an additive model (12). While in cultured HAoSMC a single copy of the deletion allele was sufficient to alter the expression of IRF2BP2 in response to LPS, *in vivo*, two copies of the deletion allele may be required to manifest the calcification phenotype.

The lack of association of the rs3045215 deletion allele with coronary calcification in CAD cases is perhaps not surprising given that atherosclerosis is the major driver of calcification in these individuals. However, the finding that men with two copies of the deletion allele have minimal burden of atherosclerosis yet moderate calcification (Agatston score of 254) would significantly increase their risk for cardiovascular events compared to men with one or no copies (Agatston scores of 110 and 113, respectively) (42). Moreover, this result suggests that calcification associated with the rs3045215 deletion may precede atherosclerosis.

Our study has several limitations. First, we do not know the consequence of the deletion allele on the response of macrophages to LPS, since our studies used THP-1 macrophages homozygous for the non-risk allele of rs3045215. Second, our study used a limited number of HAoSMC primary cultures, 3 from homozygous non-risk donors and 2 from heterozygous donors. While all 3 homozygous non-risk donor HAoSMC showed increased IRF2BP2 levels after LPS treatment (in contrast to THP-1 macrophages), heterozygous cells showed either no response or reduced IRF2BP2 levels after LPS. Third, we have not identified the RNA-binding protein that targets the rs3045215 deleted sequence. While this protein may be related to the ELAVL1 partner protein TTP (ZFP36), there is a prohibitive number of RNA-binding protein candidates to test (including ELAVL2, ELAVL3, ELAVL4, ZFP36, ZFP36L1, ZFP36L2, and among others) (43). While the 9 nucleotide rs3045215 deletion might disrupt a micro-RNA binding target or a putative alternative polyadenylation site, these are unlikely mechanisms. First, while the rs3045215 deletion lies immediately adjacent to a conserved target of the miR-17–93 cluster of micro-RNAs (http://www.targetscan.org/) it does not actually disrupt the consensus sequence and is unlikely to affect regulation by this mechanism. Second, IRF2BP2 is one of many genes that select proximal alternative polyadenylation sites to shorten their mRNAs upon immune activation, reducing the available micro-RNA targets in their mRNAs (44). However, the rs3045215 deletion does not coincide with any documented alternative polyadenylation signal (https://exon.apps.wistar.org/PolyA_DB/v3/). What our study has established is that the deletion variant affects how ELAVL1 modulates the IRF2BP2 3′UTR to control protein expression. Moreover, our study identified a novel association of the rs3045215 deletion with coronary artery calcification in men with a minimal burden of atherosclerosis. This finding will need to be confirmed in other geographically distinct independent CT angiography cohorts.

A previous Mendelian randomization analysis revisited the contribution of genetic variants at 23 loci contributing to atherosclerosis to ascertain whether these variants also predispose to coronary artery calcification beyond their effect on atherosclerosis (45). Of 23 loci showing genome-wide significant association with coronary atherosclerosis, only two remained genome-wide significant for coronary artery calcification when allele frequencies were compared between individuals with atherosclerosis with minimal calcification to individuals with calcified atherosclerosis. One was the well-known locus at 9p21.3 and the other was at the PHACTR1 locus. Thus, these loci contribute not only to atherosclerosis, but also to the process of coronary artery calcification beyond that which associates with atherosclerosis. The mechanisms whereby these loci contribute to calcification remain unknown.

Single gene mutations are known to cause arterial calcification independent of atherosclerosis. For example, mutations in the ectonucleotide pyrophosphatase/ phosphodiesterase 1 (ENPP1) gene cause generalized arterial calcification of infancy (46), due to a failure to produce pyrophosphate required to inhibit hydroxyapatite crystal growth in the vessel wall. It is intriguing that heterozygous carriers of ENPP1 mutations are prone to osteoporosis (47). On the other hand, loss of ABCC6, the transporter that carries ATP outside the cell required for pyrophosphate generation causes pseudoxanthoma elasticum, a condition associated with peripheral artery calcification (48, 49) that does not appear to predispose to osteoporosis (50). Thus, mechanisms that cause arterial calcification may not always contribute to osteoporosis. Similar to what we have reported here, a genetic variant near the gene encoding the matrix GLA protein (MGP) that also inhibits hydroxyapatite crystal growth has been tied to osteoporosis (25, 26) and is in linkage disequilibrium with a genetic variant in the MGP 5′-UTR tied to arterial calcification independent of atherosclerosis (51). Over 500 loci show genome-wide significant association with osteoporosis (25). It will be interesting to determine whether any of these osteoporosis risk alleles increase coronary artery calcification in CT angiography cohorts randomized by the degree of atherosclerotic burden, as we have done here. Some of these osteoporosis risk variants may contribute to coronary artery calcification either with or without atherosclerosis.

Recent studies have shown that IRF2BP2 activates KLF2 and plays a central role in bone formation by suppressing osteoclasts and promoting osteoblast differentiation (52). IRF2BP2 also works as a corepressor of NFAT1 (53) and this action may limit the NFAT1-dependent activation of osteoclasts. Thus, reduced expression of IRF2BP2 tied to the rs3045215 deletion may explain why individuals who carry the co-inherited SNP rs6672925 have increased susceptibility to osteoporosis. Here, we found that ELAVL1 plays an important part in mediating differential expression of IRF2BP2 by suppressing mRNAs carrying the rs3045215 deletion allele. ELAVL1 knockdown increases osteogenic differentiation and the mRNA levels of genes controlling the extracellular matrix (54). ELAVL1 also protects against non-alcoholic fatty liver disease (NAFLD) (55), a condition that associates with increased risk of coronary artery calcification (56). NAFLD is made worse by ablation of Irf2bp2 in the mouse (57). Together with recent work tying ELAVL1 in mediating the pro-atherosclerosis effect of a macrophage-specific lncRNA (24), these studies point to an interesting parallel that may contribute to arterial calcification mediated through ELAVL1 modulation of IRF2BP2.

## Data Availability Statement

All data are available in the main text or the Supplemental Material. Only summary patient data and genotypes are provided to protect the confidentiality of study participants. Data are available upon request.

## Ethics Statement

The studies involving human participants were reviewed and approved by the Ottawa Health Science Network Research Ethics Board (OHSN-REB). The patients/participants provided their written informed consent to participate in this study.

## Author Contributions

RV and AD obtained and analyzed the data. RV, AD, FS, H-HC, and AS wrote the manuscript. H-HC and AS obtained research funding. All authors contributed to the article and approved the submitted version.

## Funding

AS and H-HC are supported by operating grants from the Canadian Institutes of Health Research (376403, H-HC; 376503, AS), Discovery grants from the Natural Sciences and Engineering Research Council of Canada (RGPIN-2019-03942, H-HC; RGPIN-2016-04985, AS).

## Conflict of Interest

The authors declare that the research was conducted in the absence of any commercial or financial relationships that could be construed as a potential conflict of interest.

## Publisher's Note

All claims expressed in this article are solely those of the authors and do not necessarily represent those of their affiliated organizations, or those of the publisher, the editors and the reviewers. Any product that may be evaluated in this article, or claim that may be made by its manufacturer, is not guaranteed or endorsed by the publisher.
